# Adult ‘PICC’ Device May be Used as a Tunnelled Central Venous Catheter in Children

**DOI:** 10.1007/s00270-017-1860-5

**Published:** 2018-01-17

**Authors:** Brooke T. Lawson, Ian A. Zealley

**Affiliations:** Department of Radiology, Ninewells Hospital, Dundee, DD1 9SY Scotland, UK

**Keywords:** Catheter, Central venous, Paediatrics, Interventional radiology, Complications

## Abstract

**Purpose:**

Central venous access in children, in particular small children and infants, is challenging. We have developed a technique employing adult peripherally inserted central venous catheters (PICCs) as tunnelled central venous catheters (TCVCs) in children. The principal advantage of this novel technique is that the removal technique is less complex than that of conventional cuffed TCVCs. The catheter can be removed simply by being pulled out and does not require general anaesthesia. The purpose of this study is to determine the success, safety and utility of this technique and to identify the rate of late complications. We describe the 6-year experience in our unit.

**Materials and Methods:**

Electronic and paper medical records were reviewed for consecutive paediatric patients who had a PICC device inserted as a TCVC over a 6-year period (September 2009 through July 2015). The following data were recorded—patient demographics, setting for PICC as TCVC insertion, use of ultrasound and fluoroscopy, PICC device type, early or late complications and date of and reason for removal.

**Results:**

Twenty-one PICCs were inserted as TCVCs in 19 children, all aged less than 10 years. Mean patient age at the time of placement was 3.7 years. Average patient weight was 15.7 kg. All insertions were successful with no significant immediate complications recorded. The most common indication for insertion in our patient sample was pseudo-obstruction secondary to gastrointestinal dysmotility disorder (24%), with cystic fibrosis infective exacerbation being the second most frequent diagnosis (14%). Suspected catheter-related infection led to early device removal in one case (4.8%). Inadvertent dislodgement occurred in one case (4.8%). Nineteen of the 21 devices (90.4%) lasted for the total intended duration of use.

**Conclusion:**

Using a PICC device as a TCVC in small children appears to be a safe technique, with an acceptable complication profile.

## Introduction

Intensive treatment of paediatric patients with oncological, haematological and other complex medical conditions often relies on durable venous access devices [1]. The choice of vascular access in infants and children is typically dictated by the severity of the illness and the expected duration of the proposed treatment [2]. TCVCs provide vascular access for frequent blood sampling and administration of chemotherapy agents, blood products, antibiotics and parenteral nutrition [3]. Although establishment of stable venous access has become integral to the management of many long-term illnesses [4], it is recognised that the process of attaining central venous access in children is more difficult than in adults because of the smaller vessel dimensions and the sharper, more angulated routes the subclavian and internal jugular veins make in infants [5, 6].

TCVCs are usually sited through the internal jugular vein, and after traversing through a subcutaneous tunnel in the anterior chest wall, exit the skin away from the site where they enter the vein [4]. Tunnelled femoral PICCs can be useful, particularly in preterm or very low-birth weight infants, if there has been failure to insert PICCs in other peripheral veins or if veins are too small in calibre relative to size of catheter. Nevertheless, studies have demonstrated that femoral vein groin-insertion sites are associated with higher rates of infectious complications [7, 8].

Conventional TCVCs can prove cumbersome in the paediatric population and are associated with relatively high complication rates in smaller children (< 1 year or < 10 kg) [5]. Catheters used as tunnelled central lines come in a wide range of sizes but are sometimes significantly larger than PICC devices because of the direct puncture into a larger central vein [9]. To overcome some of these technical issues, our unit has developed a technique employing adult PICC devices as TCVCs in children. The main fundamental difference between an adult PICC device and a conventional paediatric TCVC is that a PICC device lacks a Dacron cuff. A Dacron cuff mounted on the catheter scars into the subcutaneous tissues within the tunnel after several days or weeks, reducing the risk of inadvertent dislodgement and acting as a barrier to infection from the skin insertion site [10]. The principal advantage of using a PICC as TCVC in this population is that the central catheter can be removed easily in the ward or community, without needing to bring the patient back to the radiology department to dissect the cuff free from adhesions which may require general anaesthesia. PICCs are available in a large range of sizes, 2–7 French (Fr), and are available in single- or dual-lumen design [9]. The smallest PICC catheter diameters compare with the some of the smallest commercially available paediatric catheters designed for tunnelling such as the BARD Broviac^®^ 2.7 Fr single-lumen catheter.

It was hoped that the use of PICCs as TCVCs in small children would be associated with equal durability, comparable complication rate, greater convenience and possibly a better cosmetic result in relation to healing of the chest wall scar in comparison with conventional cuffed central devices. By durability, we wanted to ascertain whether catheters lasted for the total *intended* duration of use and remained in situ until no longer required

The purpose of this study is to determine the success, safety and utility of this novel technique, and to identify the rate of late complications. The outcome was determined as successful if the catheter was still functioning properly at the time of removal. We describe the 6-year experience in out unit.

## Materials and Methods

Appropriate institutional research approval was obtained and data gathered retrospectively from electronic and paper medical records, which were reviewed for consecutive paediatric patients who had a PICC device inserted as TCVC over a 6-year period (September 2009 through July 2015).

For each individual case, the decision to use this technique was made based on the anticipated duration of treatment. Our technique was carried out when treatment was expected to be required for longer than a few days (for which peripheral cannulas would suffice) but shorter than several months or longer (for which a portacath would be the preferred device).

The following data were recorded—patient demographics, setting for PICC as TCVC insertion, use of ultrasound and fluoroscopy, PICC device type, site of surgical insertion, early or late complications and date of and reason for removal. CVC-related complications can be divided into early complications (mechanical and infective) and late complications (mechanical and infective). Early complications are generally secondary to the insertion procedure. Complications were defined as early if they occurred in the first week after the CVC insertion; all complications occurring thereafter were defined as late complications [11].

Microbiology results were reviewed to identify any laboratory-confirmed catheter-related infections. Cases in which complications or misadventure resulted in premature removal of the catheter were recorded. Data were entered into an Excel™ spread sheet and analysed using basic Excel™ statistical tools.

### Technique

**Fig. 1:**
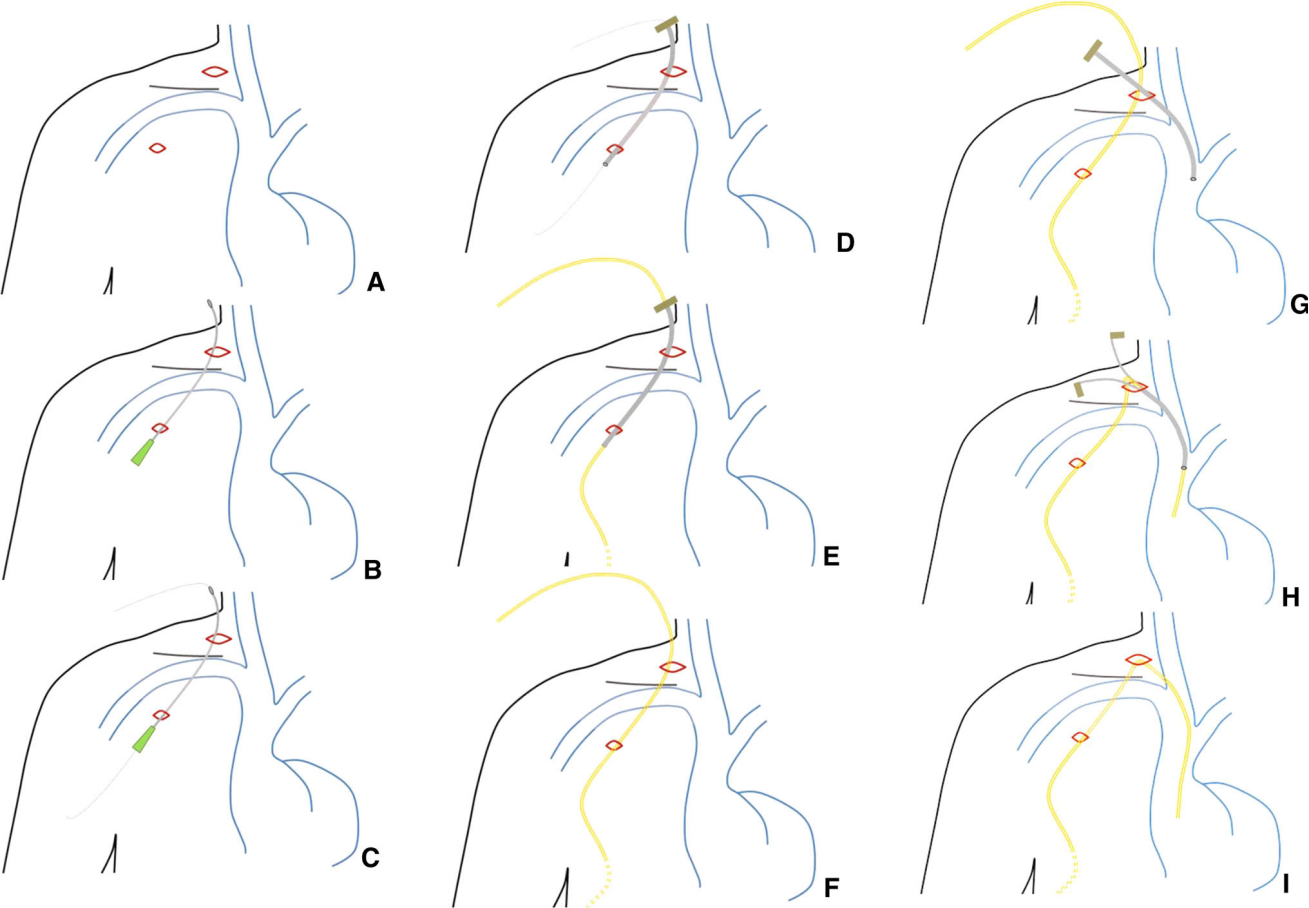
Illustrations demonstrating our novel technique, step-by-step. **A** Local anaesthetic is infiltrated from the RIJV incision site to the RACW exit site, situated approximately midway between the nipple and axilla, along a 1–2 inch track below which the subcutaneous tunnel will be fashioned. A small 5 mm RIJV site incision and a smaller 2 mm RACW site incision are made. **B** The venous access needle is tunnelled from the RACW incision to the RIJV entry site incision. **C** A 0.018-inch guidewire is passed through the access needle. **D** A 5 Fr peel-away sheath is then passed over the guidewire from the RIJV entry site to the RACW exit site. **E** The stylet and guidewire are removed, and a PICC device is passed through the peel-away sheath from the RACW site to the RIJV site. **F** The peel-away sheath is then withdrawn off the PICC and reassembled with the stylet for subsequent use. **G** The RIJV is then cannulated with a 21 Fr needle under ultrasound guidance, and a 0.018-inch guidewire is passed into the right atrium, after which the peel-away sheath assembly is advanced over the guidewire into the RIJV. **H** The PICC is advanced through the peel-away sheath into the RIJV, and the catheter is cut to appropriate length using fluoroscopic guidance in the same manner as conventional TCVCs, after which the guidewire and stylet are removed and the PICC device introduced down the peel-away sheath into the central veins. **I** The peel-away sheath is subsequently removed

**Fig. 2:**
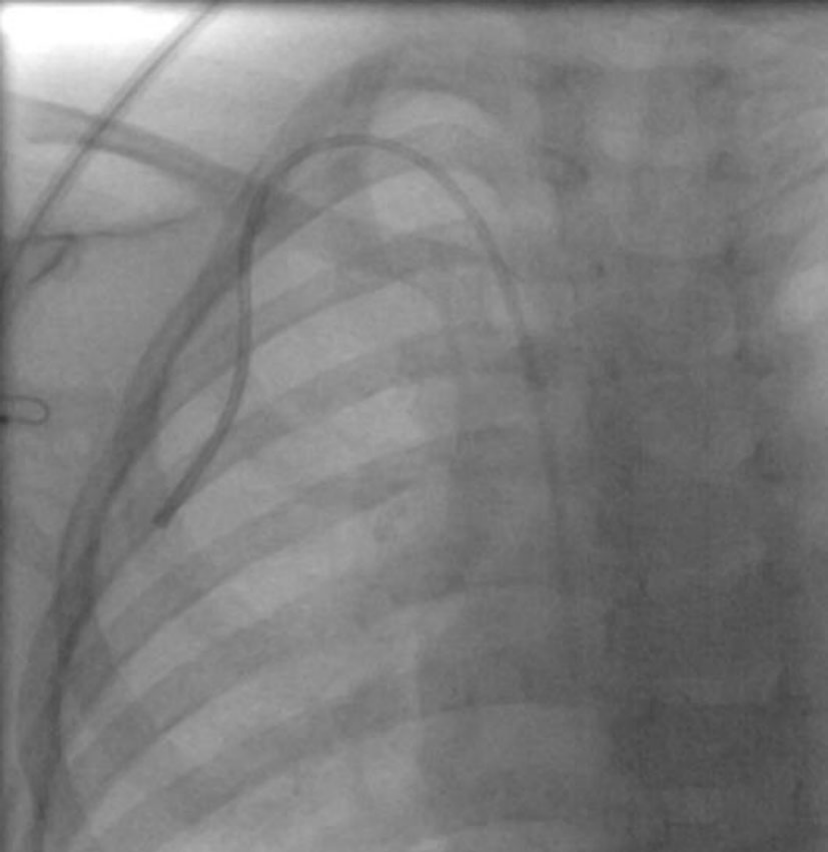
Fluoroscopic image demonstrating the tip of the PICC used as a TCVC at the superior vena cava-right atrium junction. We typically aim for catheter position in the right atrium

**Fig. 3:**
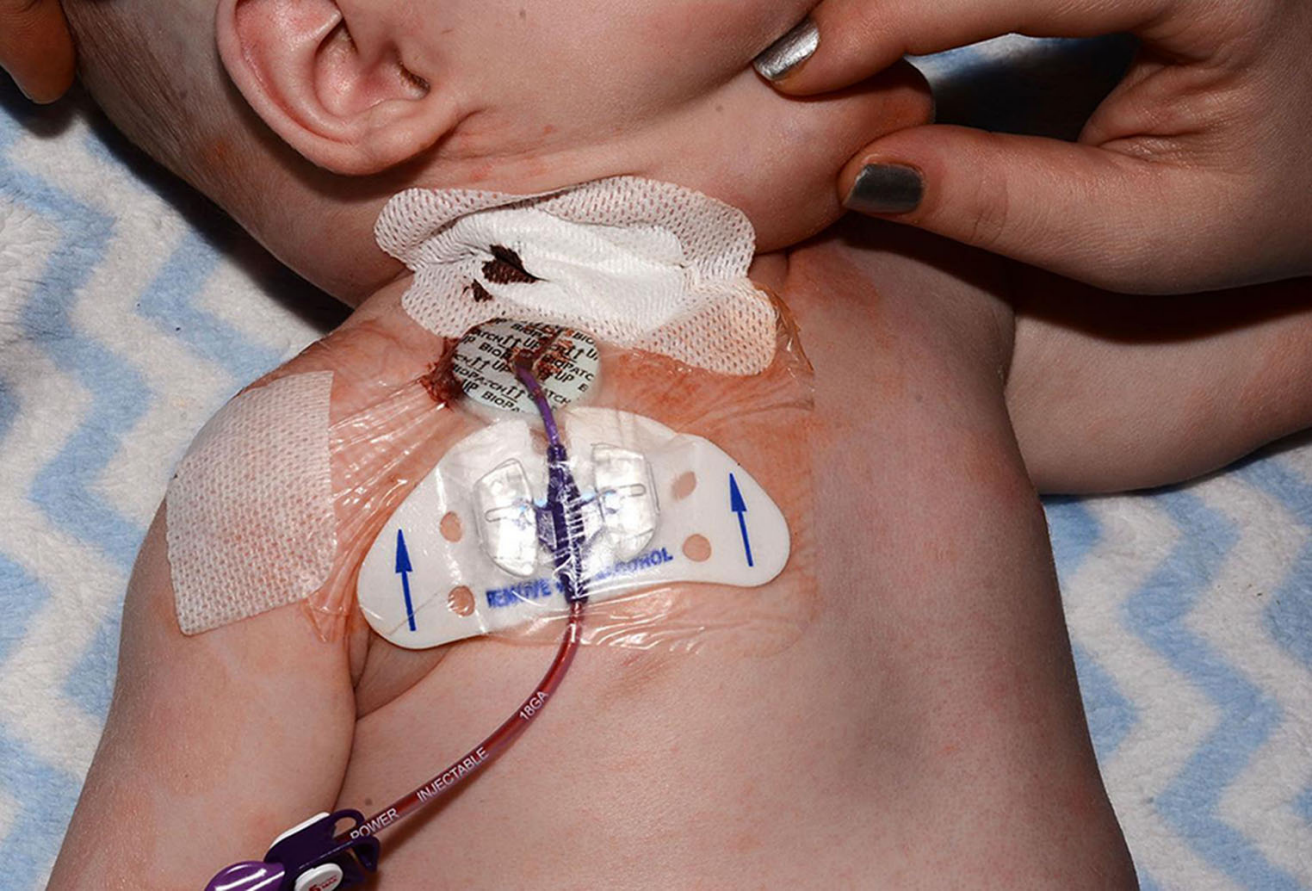
Post-operative photograph illustrating how the PICC device is secured using the proprietary adhesive dressing supplied with the device, applied to the RACW. Skin incision closure is achieved with adhesive steristrips

A consultant interventional radiologist carried out all procedures using an aseptic technique. The preferred site for access is one of the internal jugular veins, usually the right. The sizes of adult PICCs used ranged from 3 to 5 Fr catheters (*MedComp*^®^/Pro-PICC^®^CT, Mexico). Figure 1 illustrates the details of the procedure step-by-step. Local anaesthetic is infiltrated from the right internal jugular vein (RIJV) incision site to a right anterior chest wall (RACW) exit site. A 21-gauge (G) access needle is passed subcutaneously from the RACW site to the RIJV site. After needle access along the tunnel track has been achieved, a 0.018-inch guidewire is passed through the access needle, which is then withdrawn. A peel-away sheath/stylet for the PICC is advanced along the guidewire in the reverse direction from the RIJV incision to the RACW incision. A PICC device is passed through the peel-away sheath. The peel-away sheath is withdrawn intact and reassembled with stylet for later use. The PICC is advanced through the peel-away sheath/stylet subsequently introduced into the RIJV after central venous access is secured using ultrasound guidance. The catheter is cut to length using fluoroscopic guidance (Fig. 2). The peel-away sheath is removed. Skin closure over the RIJV incision site is achieved with steristrips, and a proprietary adhesive securing device is used at the RACW incision site (Fig. 3).

## Results

**Fig. 4:**
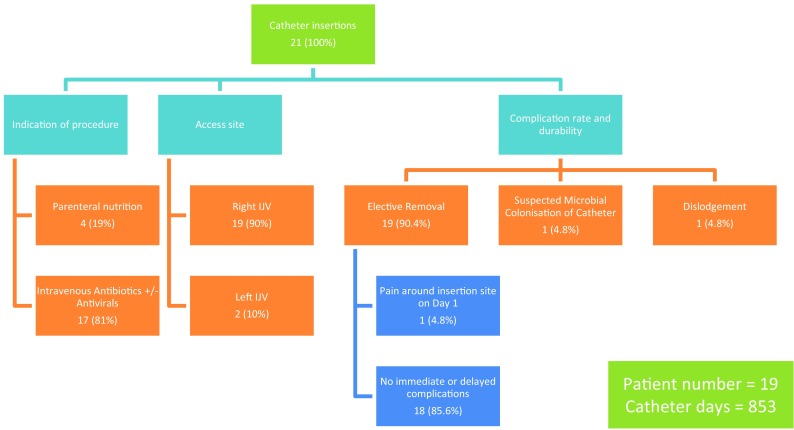
Procedural data, complication rate and durability

**Fig. 5:**
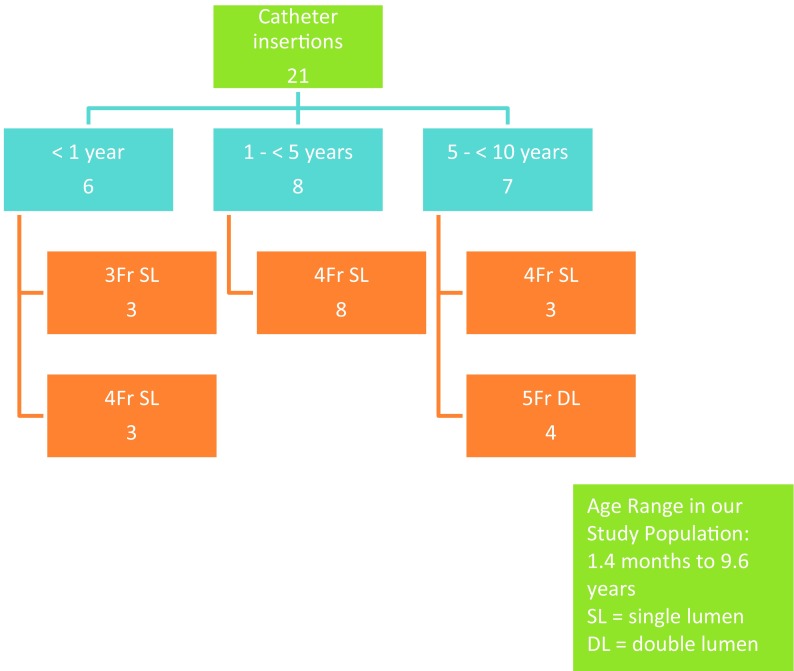
Catheter size versus patient age

Twenty-one PICCs were inserted as TCVCs in 19 children, all aged less than 10 years. Mean patient age at the time of placement was 3.7 years (range 1.4 months–9.6 years). Five patients (24%) were less than 1 year of age or less than 10 kg in weight. Average patient weight was 15.7 kg. The most frequent underlying patient conditions that precipitated the indication for long-term central venous access was pseudo-obstruction secondary to gastrointestinal dysmotility disorder in five patients (24%) followed by cystic fibrosis infective exacerbation in three patients (14%). Specific indications were for the administration of parenteral nutrition in 4/21 cases (19%) and intravenous antibiotics and/or antiviral therapy in 17/21 cases (81%). Figure 4 summarises the indication for catheter insertion. The total number of catheter days reviewed was 853. Catheter dwell time ranged from 6 days to 6 months with a mean catheter dwell time of 41 days. Catheter devices used included 3 Fr single lumen in three cases (14%), 4 Fr single-lumen catheters in 14 cases (67%) and 5 Fr dual-lumen catheters in four cases (19%). Figure 5 summarises catheter size versus patient age at insertion. General anaesthesia and local anaesthesia were used for all catheter insertions (100%). The procedure was carried out under ultrasound and fluoroscopic guidance for 18 (86%) cases, all in the interventional radiology (IR) suite, and ultrasound only for three cases (14%), all in the paediatric operating room (OR) suite. Subsequent conventional X-ray confirmation of tip location was obtained in the later cases. The RIJV was the access site in 19/21 insertions (90%). The left internal jugular vein (LIJV) was chosen as the access site in two cases (10%) when there had been prior damage to the RIJV access site caused by prior venous access procedures. Figure 4 also summarises the access site used in all procedures.

All insertions were successful with no significant immediate or early complications recorded. In our population, premature catheter removal occurred in two cases (9.6%) with an overall late complication rate of 2.3 per 1000 catheter days. Inadvertent catheter dislodgement occurred in one case (4.8%) at 10 days post-insertion (dislodgement rate of 1.2 per 1000 catheter days). This case was a 3-year-old male receiving intravenous antibiotics for osteomyelitis. We suspect that inadvertent dislodgement occurred during a change of clothes by the child’s parents.

Early catheter removal at 14 days was performed in one case (4.8%) for suspected catheter-related infection (suspected catheter-related infection rate of 1.2 per 1000 catheter days). This case was a 9-year-old female with Congenital Myotonic Dystrophy who had a gastrostomy and colostomy for bowel dysfunction. Pseudo-obstruction precipitated central venous access to facilitate administration of parenteral nutrition. On day 11, a temperature spike led to blood cultures from the catheter which grew a strain of *Staphylococcus aureus*, with which the stoma and gastrostomy sites were known to be colonised prior to catheter insertion.

One patient (4.8%) experienced pain around the insertion site on the day following central venous access insertion: this resolved with simple analgesia and did not necessitate premature catheter removal.

Figure 4 summarises the procedural data and overall outcomes. Nineteen of the 21 TCVCs (90.4%) lasted for the total intended duration of use.

## Discussion

Demand for radiologically inserted vascular access devices in children is increasing.

The most common paediatric vascular access device inserted by an interventional radiologist is the PICC. However, according to Krishnamurthy et al. [4], TCVCs last longer than PICCs and are preferred when access is required for more than 6 weeks duration. In a recent retrospective cohort study conducted by Kovacich et al. [12] looking at PICC-associated complications in children requiring long-term parenteral antibiotic therapy, there was an overall complication rate of 4.6 per 1000 catheter days, with catheter occlusion and dislodgement being the most common reasons for premature PICC removal. On the other hand, there are many institutions that use PICC devices for long-term paediatric venous access, and there are some data to support PICC devices having fewer complications than TCVCs. Blotte et al. [13] carried out a retrospective analysis comparing the complications of Broviacs^®^ TCVC and PICCs in children with intestinal failure receiving parenteral nutrition. When comparing catheters with the same diameter, there were no significant differences in infection or breakage rates. However, a lower incidence of central venous thrombosis with the use of PICCs is suggested. This correlates with evidence in the literature, where risk factors for central venous thrombosis include catheter size, location of the catheter tip and associated catheter complications. Another prospective randomised study by Cowl et al. [14] found no difference in rates of infection, occlusion or dislodgement when comparing PICCs with subclavian central catheters.

However, their use is associated with frequent complications resulting in premature catheter removal [1]. Infectious complications include exit site or port infection, tunnel infection and microbial colonisation of the catheter (defined by either positive culture from the CVC with negative peripheral blood culture, or positive catheter tip culture). Mechanical complications include inadvertent dislodgement, catheter fracture, occlusion and venous thrombosis [3]. Image guidance has been found to increase procedure success rate and decrease acute complication rates.

The 4.8% incidence (1.2 per 1000 catheter days) of suspected microbial colonisation reported in our study is in the lower range of reported rates in the literature. Garcia-Teresa et al. [15] in a multicentre prospective study examining children in a paediatric intensive care unit (ICU) aged 0–14 years report a catheter-related blood stream infection rate of 6.81% or 6.4 per 1000 catheter days. Casado-Flores e al. [16] conducted a prospective study looking at central venous catheterisations in children of different ages in a paediatric ICU and found an infection rate of 5.8%. Cruzeiro et al. [17] in a prospective study of consecutive catheterisations in children in a public hospital report an 11.6% of suspected catheter-related infection.

Multiple studies have also been carried out looking at complication rates of CVCs in neonates, with infection rates varying from 0 to 46% [1, 18–27]. Battin et al. [27] recently conducted a prospective audit assessing complication rates in a neonatal ICU. In total, 38% of babies showed clinical signs of sepsis while their lines were in situ but only 10% had positive peripheral or line cultures. On the other hand, Ainsworth et al. [18] recently conducted a meta-analysis looking at randomised controlled trials that compared delivery of intravenous fluids via CVCs versus peripheral cannulae in hospitalised neonates. In conclusion, there was no evidence to suggest that percutaneous CVC use increases risks of adverse events, particularly invasive infection.

Dislodgement occurred in only one case (4.8%) in this series, with a rate of dislodgement of 1.2 per 1000 catheter days. We suspect that inadvertent dislodgement occurred during a change of clothes by the child’s parents. The current study identified a rate of dislodgement in the lower range of that reported in previous studies. Central venous catheter dislodgement has been found to be more frequent in younger patients [1]. A retrospective study by Tavis et al. [3] comparing delayed complications of surgically versus radiologically placed CVCs in paediatric oncology patients quotes a rate of dislodgement of 16.7% amongst radiologically placed CVCs. Nosher et al. [28] examining a sample of paediatric CVCs predominantly placed for chemotherapy reported rates of dislodgment at 12% (0.82 per 1000 catheter days). Wiener et al. [29] in a large, multicentre study combining data from ports and CVCs placed for chemotherapy in children reported rates of dislodgement ranging from 2.8 to 24%. This suggests that despite lacking a Dacron cuff and with only a proprietary adhesive anchoring mechanism in place, these non-cuffed devices are reasonably secure. We attribute this infrequent rate of dislodgement to the fact that these tunnelled catheters can be tucked away safely under the child’s clothing and are less likely to get accidentally pulled out in comparison with peripherally inserted catheters.

The results not only indicate our technique to be safe with an acceptable low complication profile, but also offer an advantage of greater convenience in comparison with conventional paediatric TCVCs. Ninety per cent of these catheters lasted for the total *intended* duration of use and remained in situ until no longer required. It is not clear what is responsible for the apparent security of the device, but experience with this model and brand of adhesive device in adult PICCs suggests that the adhesive device alone provides durable device retention without suturing or the presence of a subcutaneous retention cuff. It is, however, recommended that the adhesive device be replaced expertly when indicated by the state of the dressings. Catheter removal in our technique is less complex. The catheter can be removed simply by pulling it out, and this does not require general anaesthesia because it does not cause any discomfort. Another advantage over standard PICCs is that the device gets tucked away safely under clothing, away from inquisitive fingers. The limitations of the current study include the retrospective non-randomized study design and the modest sample size.

Future prospective studies comparing this novel technique with standard PICCs and conventional TCVCs placed over a similar time period would be of value to confirm equal utility, comparable complication rate and possibly a better cosmetic result in relation to healing of the chest wall scar. Although the subgroup of five patients (24%) who were less than 1 year of age or less than 10 kg in weight were not analysed separately, none of these patients sustained delayed complications in our study population. To the best of our knowledge, although we are aware anecdotally of other units employing similar techniques, we are not aware of any priorly published report describing this simple technique.

## Conclusion

Using an adult PICC device as a TCVC in infants and children, including children less than 1 year of age or weighing less than 10 kg, appears to be a safe technique with an acceptable complication profile. The principal advantage of this technique is that the catheter removal technique is less complex than that of standard TCVC. The catheter can be removed simply by pulling it out, and this does not require general anaesthesia.
